# Supplementary material for: Determining the influence of LPI, GCI and IR on FDI: A study on the Asia and Pacific Region
Source: PLoS One. 2023 Feb 1;18(2):e0281246. doi: 10.1371/journal.pone.0281246 (PMC9891520; doi:10.1371/journal.pone.0281246)
Supplement: S2 Appendix — (DOCX) [file pone.0281246.s002.docx]

**S2 Appendix. Summary descriptive statistics of variables**

| **Continents** | | **Variables** | | | | **Continents** | | **Variables** | | | |
| --- | --- | --- | --- | --- | --- | --- | --- | --- | --- | --- | --- |
|  |  | **FDI  ($ bn)** | **GCI** | **LPI** | **IR** |  |  | **FDI  ($ bn)** | **GCI** | **LPI** | **IR** |
| Asia &  Pacific | Obs. | 177 | 177 | 177 | 177 | Korea,  Rep. | Obs. | 5 | 5 | 5 | 5 |
|  | Mean | 69.50 | 4.455 | 3.043 | 4.383 |  | Mean | 10.500 | 5.111 | 3.666 | 2.928 |
|  | SD | 306.00 | 0.620 | 0.541 | 5.902 |  | SD | 1.490 | 0.238 | 0.042 | 1.012 |
|  | Min. | -4.16 | 3.310 | 1.933 | -13.730 |  | Min. | 9.270 | 4.930 | 3.612 | 1.355 |
|  | Max. | 2590.00 | 5.845 | 4.190 | 24.270 |  | Max. | 12.200 | 5.516 | 3.717 | 4.093 |
| Armenia | Obs. | 6 | 6 | 6 | 6 | Kuwait | Obs. | 6 | 6 | 6 | 6 |
|  | Mean | 0.450 | 3.967 | 2.452 | 12.524 |  | Mean | 0.841 | 4.491 | 3.020 | 2.286 |
|  | SD | 0.145 | 0.177 | 0.224 | 2.647 |  | SD | 1.100 | 0.094 | 0.172 | 8.383 |
|  | Min. | 0.267 | 3.750 | 2.137 | 9.734 |  | Min. | -0.021 | 4.347 | 2.830 | -6.890 |
|  | Max. | 0.668 | 4.193 | 2.673 | 17.042 |  | Max. | 2.870 | 4.590 | 3.281 | 12.145 |
| Australia | Obs. | 6 | 6 | 6 | 6 | Kyrgyz  Republic | Obs. | 6 | 6 | 6 | 6 |
|  | Mean | 50.80 | 5.219 | 3.786 | 4.634 |  | Mean | 0.341 | 3.572 | 2.372 | 12.777 |
|  | SD | 11.50 | 0.167 | 0.041 | 1.290 |  | SD | 0.178 | 0.184 | 0.182 | 3.572 |
|  | Min. | 35.20 | 5.080 | 3.727 | 3.032 |  | Min. | 0.144 | 3.310 | 2.156 | 7.262 |
|  | Max. | 63.20 | 5.523 | 3.841 | 6.041 |  | Max. | 0.619 | 3.750 | 2.619 | 17.427 |
| Azerbaijan | Obs. | 4 | 4 | 4 | 4 | Lebanon | Obs. | 5 | 5 | 5 | 5 |
|  | Mean | 4.420 | 4.323 | 2.465 | 9.838 |  | Mean | 3.100 | 3.866 | 2.816 | 5.056 |
|  | SD | 0.802 | 0.201 | 0.143 | 9.239 |  | SD | 0.695 | 0.128 | 0.298 | 3.443 |
|  | Min. | 3.350 | 4.060 | 2.291 | -1.204 |  | Min. | 2.570 | 3.680 | 2.580 | -0.287 |
|  | Max. | 5.290 | 4.530 | 2.640 | 19.446 |  | Max. | 4.280 | 4.039 | 3.338 | 7.713 |
| Bahrain | Obs. | 4 | 4 | 4 | 4 | Malaysia | Obs. | 6 | 6 | 6 | 6 |
|  | Mean | 1.240 | 4.483 | 3.165 | 2.566 |  | Mean | 10.20 | 5.096 | 3.442 | 2.035 |
|  | SD | 0.733 | 0.148 | 0.144 | 3.702 |  | SD | 1.89 | 0.117 | 0.123 | 2.276 |
|  | Min. | 0.156 | 4.280 | 3.053 | -0.180 |  | Min. | 8.30 | 4.880 | 3.221 | -2.113 |
|  | Max. | 1.760 | 4.630 | 3.373 | 7.667 |  | Max. | 13.50 | 5.208 | 3.590 | 4.226 |
| Bangladesh | Obs. | 5 | 5 | 5 | 5 | Mongolia | Obs. | 6 | 6 | 6 | 6 |
|  | Mean | 1.840 | 3.653 | 2.604 | 4.940 |  | Mean | 0.745 | 3.763 | 2.303 | 6.103 |
|  | SD | 0.845 | 0.126 | 0.103 | 1.413 |  | SD | 2.800 | 0.104 | 0.143 | 10.526 |
|  | Min. | 0.651 | 3.460 | 2.471 | 3.449 |  | Min. | -4.160 | 3.600 | 2.085 | -13.730 |
|  | Max. | 2.540 | 3.800 | 2.743 | 6.886 |  | Max. | 4.270 | 3.870 | 2.506 | 17.131 |
| Brunei | Obs. | 2 | 2 | 2 | 2 | New  Zealand | Obs. | 6 | 6 | 6 | 6 |
|  | Mean | 0.183 | 4.324 | 2.789 | 6.372 |  | Mean | 2.700 | 5.183 | 3.621 | 3.004 |
|  | SD | 0.471 | 0.037 | 0.116 | 13.826 |  | SD | 1.470 | 0.175 | 0.188 | 2.818 |
|  | Min. | -0.151 | 4.298 | 2.707 | -3.404 |  | Min. | 0.286 | 4.920 | 3.388 | -1.995 |
|  | Max. | 0.516 | 4.350 | 2.870 | 16.148 |  | Max. | 4.340 | 5.425 | 3.876 | 5.974 |
| China | Obs. | 6 | 6 | 6 | 6 | Oman | Obs. | 5 | 5 | 5 | 5 |
|  | Mean | 220.00 | 4.805 | 3.521 | 1.761 |  | Mean | 2.42 | 4.502 | 3.031 | 1.655 |
|  | SD | 44.00 | 0.292 | 0.116 | 2.230 |  | SD | 2.01 | 0.145 | 0.178 | 9.105 |
|  | Min. | 156.00 | 4.240 | 3.322 | -1.002 |  | Min. | 1.24 | 4.280 | 2.839 | -7.346 |
|  | Max. | 268.00 | 5.082 | 3.661 | 4.522 |  | Max. | 5.94 | 4.650 | 3.234 | 15.278 |
| Egypt | Obs. | 6 | 6 | 6 | 6 | Pakistan | Obs. | 6 | 6 | 6 | 6 |
|  | Mean | 6.940 | 3.804 | 2.823 | -0.124 |  | Mean | 2.450 | 3.525 | 2.690 | 5.410 |
|  | SD | 3.070 | 0.188 | 0.291 | 4.346 |  | SD | 1.640 | 0.084 | 0.198 | 2.074 |
|  | Min. | 2.800 | 3.600 | 2.371 | -6.263 |  | Min. | 0.859 | 3.420 | 2.419 | 2.880 |
|  | Max. | 11.600 | 4.070 | 3.185 | 6.922 |  | Max. | 5.590 | 3.660 | 2.923 | 8.321 |
| Georgia | Obs. | 5 | 5 | 5 | 5 | Philippines | Obs. | 6 | 6 | 6 | 6 |
|  | Mean | 1.330 | 4.147 | 2.538 | 7.863 |  | Mean | 5.20 | 4.216 | 2.937 | 3.522 |
|  | SD | 0.409 | 0.185 | 0.162 | 4.690 |  | SD | 3.42 | 0.192 | 0.157 | 1.174 |
|  | Min. | 0.921 | 3.860 | 2.353 | 1.450 |  | Min. | 1.07 | 3.960 | 2.689 | 2.292 |
|  | Max. | 1.840 | 4.320 | 2.774 | 14.278 |  | Max. | 9.95 | 4.400 | 3.142 | 5.358 |
| Hong  Kong | Obs. | 6 | 6 | 6 | 6 | Qatar | Obs. | 6 | 6 | 6 | 6 |
|  | Mean | 1540.00 | 5.479 | 3.970 | 2.858 |  | Mean | 1.57 | 5.078 | 3.306 | 1.737 |
|  | SD | 753.00 | 0.153 | 0.115 | 1.465 |  | SD | 2.68 | 0.294 | 0.281 | 7.537 |
|  | Min. | 446.00 | 5.300 | 3.827 | 1.335 |  | Min. | -2.19 | 4.550 | 2.950 | -6.613 |
|  | Max. | 2590.00 | 5.761 | 4.121 | 4.717 |  | Max. | 4.70 | 5.380 | 3.599 | 14.301 |
| India | Obs. | 6 | 6 | 6 | 6 | Russian  Federation | Obs. | 6 | 6 | 6 | 6 |
|  | Mean | 33.00 | 4.360 | 3.156 | 4.102 |  | Mean | 35.50 | 4.332 | 2.597 | 0.955 |
|  | SD | 8.83 | 0.107 | 0.135 | 3.330 |  | SD | 17.90 | 0.195 | 0.133 | 4.838 |
|  | Min. | 24.00 | 4.210 | 3.071 | -1.984 |  | Min. | 8.78 | 4.080 | 2.368 | -3.345 |
|  | Max. | 44.50 | 4.520 | 3.420 | 6.695 |  | Max. | 55.90 | 4.592 | 2.757 | 9.484 |
| Indonesia | Obs. | 6 | 6 | 6 | 6 | Singapore | Obs. | 6 | 6 | 6 | 6 |
|  | Mean | 15.30 | 4.454 | 2.989 | 5.139 |  | Mean | 62.90 | 5.666 | 4.092 | 3.465 |
|  | SD | 8.13 | 0.116 | 0.134 | 4.081 |  | SD | 12.80 | 0.119 | 0.078 | 2.337 |
|  | Min. | 4.54 | 4.260 | 2.760 | -1.746 |  | Min. | 47.30 | 5.480 | 3.996 | -0.553 |
|  | Max. | 25.10 | 4.570 | 3.150 | 9.224 |  | Max. | 83.10 | 5.845 | 4.190 | 5.635 |
| Iran | Obs. | 3 | 3 | 3 | 3 | Sri Lanka | Obs. | 5 | 5 | 5 | 5 |
|  | Mean | 3.890 | 4.160 | 2.555 | 0.763 |  | Mean | 0.906 | 4.084 | 2.546 | 1.374 |
|  | SD | 0.679 | 0.053 | 0.058 | 13.783 |  | SD | 0.441 | 0.175 | 0.197 | 6.817 |
|  | Min. | 3.370 | 4.120 | 2.489 | -10.496 |  | Min. | 0.478 | 3.870 | 2.288 | -10.246 |
|  | Max. | 4.660 | 4.220 | 2.601 | 16.134 |  | Max. | 1.610 | 4.250 | 2.750 | 7.443 |
| Israel | Obs. | 3 | 3 | 3 | 3 | Tajikistan | Obs. | 6 | 6 | 6 | 6 |
|  | Mean | 13.20 | 5.164 | 3.410 | 2.476 |  | Mean | 0.247 | 3.756 | 2.249 | 13.640 |
|  | SD | 7.80 | 0.206 | 0.218 | 0.354 |  | SD | 0.093 | 0.242 | 0.215 | 10.799 |
|  | Min. | 6.05 | 4.950 | 3.260 | 2.192 |  | Min. | 0.094 | 3.500 | 1.933 | -3.362 |
|  | Max. | 21.50 | 5.362 | 3.660 | 2.873 |  | Max. | 0.360 | 4.120 | 2.527 | 24.270 |
| Japan | Obs. | 5 | 5 | 5 | 5 | Thailand | Obs. | 6 | 6 | 6 | 6 |
|  | Mean | 18.100 | 5.464 | 3.961 | 1.699 |  | Mean | 9.65 | 4.606 | 3.313 | 2.478 |
|  | SD | 15.500 | 0.089 | 0.042 | 1.603 |  | SD | 4.69 | 0.084 | 0.096 | 1.268 |
|  | Min. | 0.547 | 5.370 | 3.915 | -0.459 |  | Min. | 3.49 | 4.510 | 3.176 | 0.243 |
|  | Max. | 41.000 | 5.600 | 4.024 | 3.546 |  | Max. | 14.70 | 4.725 | 3.430 | 3.487 |
| Jordan | Obs. | 6 | 6 | 6 | 6 | Vietnam | Obs. | 6 | 6 | 6 | 6 |
|  | Mean | 1.760 | 4.230 | 2.784 | 4.529 |  | Mean | 9.58 | 4.146 | 3.044 | 3.185 |
|  | SD | 0.576 | 0.047 | 0.150 | 2.252 |  | SD | 2.48 | 0.156 | 0.143 | 1.940 |
|  | Min. | 0.955 | 4.151 | 2.557 | 0.876 |  | Min. | 6.70 | 3.890 | 2.889 | 0.947 |
|  | Max. | 2.620 | 4.290 | 2.957 | 6.834 |  | Max. | 12.60 | 4.310 | 3.274 | 5.785 |

Note: Obs. – Observations, SD – Standard Deviation, Min. – Minimum value, Max. – Maximum value
