# Supplementary material for: Determining the influence of LPI, GCI and IR on FDI: A study on the Asia and Pacific Region
Source: PLoS One. 2023 Feb 1;18(2):e0281246. doi: 10.1371/journal.pone.0281246 (PMC9891520; doi:10.1371/journal.pone.0281246)
Supplement: S3 Appendix — (DOCX) [file pone.0281246.s003.docx]

| **Variable** | **Asia & Pacific** | **Armenia** | **Australia** | **Bangladesh** | **China** | **Egypt** | **Georgia** | **Hong Kong** | **India** |
| --- | --- | --- | --- | --- | --- | --- | --- | --- | --- |
|  | **lnFDI** | **lnFDI** | **InFDI** | **InFDI** | **InFDI** | **InFDI** | **InFDI** | **InFDI** | **InFDI** |
|  | **RE** | **MLR** | **MLR** | **MLR** | **MLR** | **MLR** | **MLR** | **MLR** | **MLR** |
| LPI | 0.2281 | -0.0054 | -3.2923 | -1.7664 | -3.6670*** | -1.5346 | -0.0487 | 2.3003 | 2.1176** |
|  | (0.3495) | (0.3186) | (1.8804) | (1.9552) | (0.3142) | (1.5309) | (3.0566) | (2.2829) | (0.3802) |
| GCI | 0.9182*** | -1.7044* | -0.4475 | 4.8726 | 1.7130*** | -0.6766 | 1.5103 | -1.5711 | -1.4379* |
|  | (0.3316) | (0.5840) | (0.9524) | (1.8113) | (0.1575) | (2.7978) | (3.3791) | (1.8464) | (0.4263) |
| Interest rates | -0.0215* | -0.0024 | -0.0942 | -0.0172 | 0.0347* | 0.0852* | -0.0175 | 0.1809 | 0.0253 |
|  | (0.0095) | (0.0287) | (0.1393) | (0.2010) | (0.0111) | (0 .0282) | (0.0933) | (0.1945) | (0.0127) |
| Constant | 17.5511*** | 26.6849*** | 39.8637** | 8.0943 | 30.7165*** | 29.4819 | 14.9677 | 26.8878 | 23.6703*** |
|  | (1.8067) | 1.8085 | (6.2517) | (8.1522) | (0.5939) | (14.9747) | (20.8722) | (17.3977) | (1.6340) |
| Observations | 173 | 6 | 6 | 5 | 6 | 6 | 5 | 6 | 6 |
| No. of years | 6 | 6 | 6 | 5 | 6 | 6 | 5 | 6 | 6 |
| R^2^ | 0.3936 | 0.8271 | 0.5463 | 0.8213 | 0.9804 | 0.7141 | 0.7177 | 0.6990 | 0.8505 |

**S3 Appendix. RE and MLR model results**

Note: *Significant at 10%, ** significant at 5%, and ***significant 1% significance level.

RE denotes Random Effect model and MLR denotes Multiple Linear Regression

Parentheses indicate robust standard error

| **Variable** | **Indonesia** | **Japan** | **Jordan** | **Korea** | **Kuwait** | **Kyrgyz** | **Lebanon** | **Malaysia** | **Mongolia** | **New Zealand** |
| --- | --- | --- | --- | --- | --- | --- | --- | --- | --- | --- |
|  | **InFDI** | **InFDI** | **InFDI** | **InFDI** | **InFDI** | **InFDI** | **InFDI** | **InFDI** | **InFDI** | **InFDI** |
|  | **MLR** | **MLR** | **MLR** | **MLR** | **MLR** | **MLR** | **MLR** | **MLR** | **MLR** | **MLR** |
| LPI | 1.1196 | 68.8566 | 0.7398 | 0.6626** | -3.3395* | -1.1658 | 0.9168** | 0.3496 | 1.1215 | 2.3161 |
|  | (4.0425) | (57.1933) | (0.6848) | (0.0371) | (0.4781) | (2.5619) | (0.0212) | (0.4501) | (8.7500) | (2.3820) |
| GCI | 2.7012 | -15.5816 | 3.4594 | 0.5297*** | 16.6698** | -0.3811 | -0.6584* | 0.7567 | 3.1841 | 6.5092 |
|  | (3.2496) | (19.3034) | (2.3482) | (0 .007249 | (1.1078) | (1.7708) | (0.0546) | (0.9758) | (11.2110) | (2.4898) |
| Interest rates | -0.0702 | -1.5318 | -0.0809 | -0.1004*** | -0.0412 | 0.0561 | -0.0460** | -0.0529 | -0.0409 | 0.3930 |
|  | (0.1901) | (1.0457) | (0.0639) | (0.0012) | (0.0218) | (0.0621) | (0.0023) | (0.0360) | (0.0619) | (0.2335) |
| Constant | 8.2731 | -162.0757 | 4.9127 | 18.2254*** | -44.8280* | 22.9417 | 22.0314*** | 18.0808* | 6.5263 | -21.8549 |
|  | 20.6596 | (129.7447) | (9.8758) | (0.1693) | (4.9727) | (10.6311) | (0.1900) | (5.0900) | (30.8908) | (19.6211) |
| Observations | 6 | 5 | 6 | 5 | 5 | 6 | 5 | 6 | 5 | 6 |
| No. of years | 6 | 5 | 6 | 5 | 5 | 6 | 5 | 6 | 5 | 6 |
| R^2^ | 0.2020 | 0.7538 | 0.7036 | 0.9999 | 0.9890 | 0.2275 | 0.9993 | 0.2385 | 0.3013 | 0.7590 |

Note: *Significant at 10%, ** significant at 5%, and ***significant 1% significance level.

RE denotes Random Effect model and MLR denotes Multiple Linear Regression

Parentheses indicate robust standard error

| **Variable** | **Oman** | **Pakistan** | **Philippines** | **Qatar** | **Russia** | **Singapore** | **Sri Lanka** | **Tajikistan** | **Thailand** | **Vietnam** |
| --- | --- | --- | --- | --- | --- | --- | --- | --- | --- | --- |
|  | **InFDI** | **InFDI** | **InFDI** | **InFDI** | **InFDI** | **InFDI** | **InFDI** | **InFDI** | **InFDI** | **InFDI** |
|  | **MLR** | **MLR** | **MLR** | **MLR** | **MLR** | **MLR** | **MLR** | **MLR** | **MLR** | **MLR** |
| LPI | 5.3979** | 1.5816 | -3.0500* | -4.1573 | -0.6792 | -1.3515 | 0.8975 | -0.3535 | 2.1010 | 0.6403 |
|  | (0.3380) | (0.8557) | (0.8618) | (2.0209) | (1.9647) | (0.8015) | (2.1523) | (1.3493) | (6.1241) | (0.7269) |
| GCI | 2.8714 | 5.5960* | 3.2291** | -1.2920 | -3.6055 | 0.9689 | -0.2873 | 1.1955 | -4.3426 | 0.2807 |
|  | (0.4929) | (1.4243) | (0.6068) | (0.7596) | (1.2513) | (0.4535) | (2.9250) | (1.5835) | (7.9730) | (0.2722) |
| Interest rates | -0.0256 | -0.1744 | -0.1686 | 0.1060 | 0.0669 | 0.0184 | 0.0334 | -0.0189 | -0.0252 | 0.0727 |
|  | (0.0041) | (0.1340) | (0.1627) | (0.0779) | (0.0335) | (0.0239) | (0.0908) | (0.0315) | (0.2269) | (0.0420) |
| Constant | -7.8435 | -1.5768 | 18.0704* | 40.9464* | 41.4524*** | 24.8252** | 19.3774 | 15.8099 | 35.9668 | 19.6113 |
|  | (3.2602) | (6.3060) | (5.0661) | (6.3443) | (1.9027) | (5.6683) | (8.5070) | (6.6075) | (25.4867) | (2.0171) |
| Observations | 5 | 6 | 6 | 5 | 6 | 6 | 5 | 6 | 6 | 6 |
| No. of years | 5 | 6 | 6 | 5 | 6 | 6 | 5 | 6 | 6 | 6 |
| R^2^ | 0.9973 | 0.5028 | 0.9678 | 0.9081 | 0.9103 | 0.8903 | 0.7033 | 0.2680 | 0.2053 | 0.7496 |

Note: *Significant at 10%, ** significant at 5%, and ***significant 1% significance level.

RE denotes Random Effect model and MLR denotes Multiple Linear Regression

Parentheses indicate robust standard error
