# Supplementary figures and images for: Determining the influence of LPI, GCI and IR on FDI: A study on the Asia and Pacific Region
Source: PLoS One. 2023 Feb 1;18(2):e0281246. doi: 10.1371/journal.pone.0281246 (PMC9891520; doi:10.1371/journal.pone.0281246)

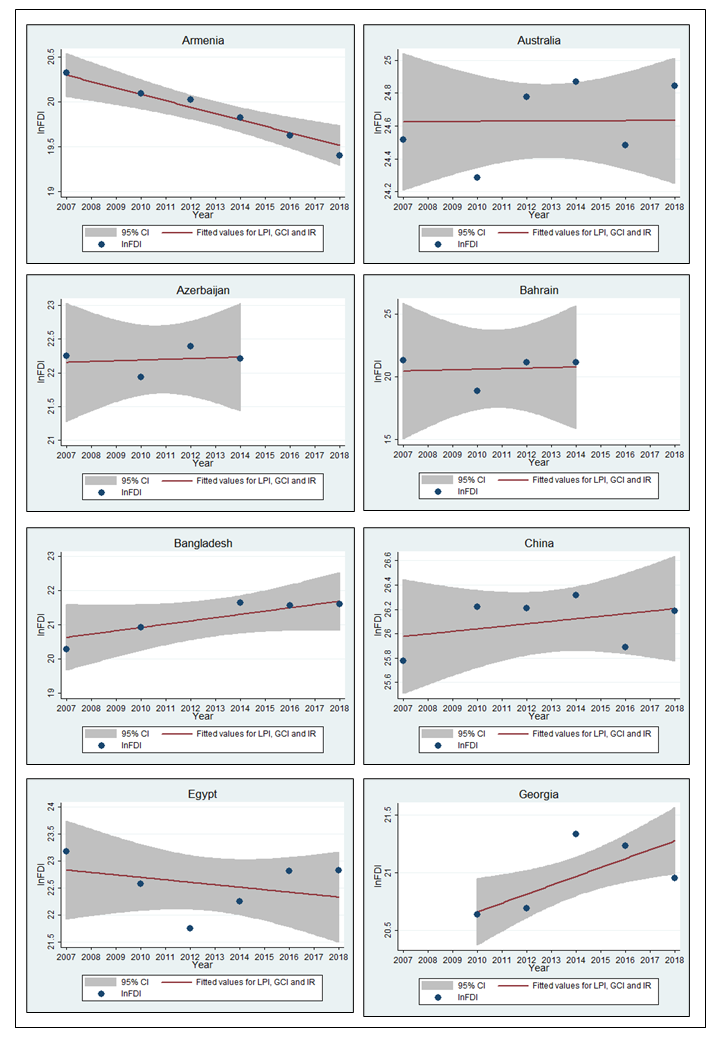
**S4 Appendix. Linear fit scatter plot graphs**


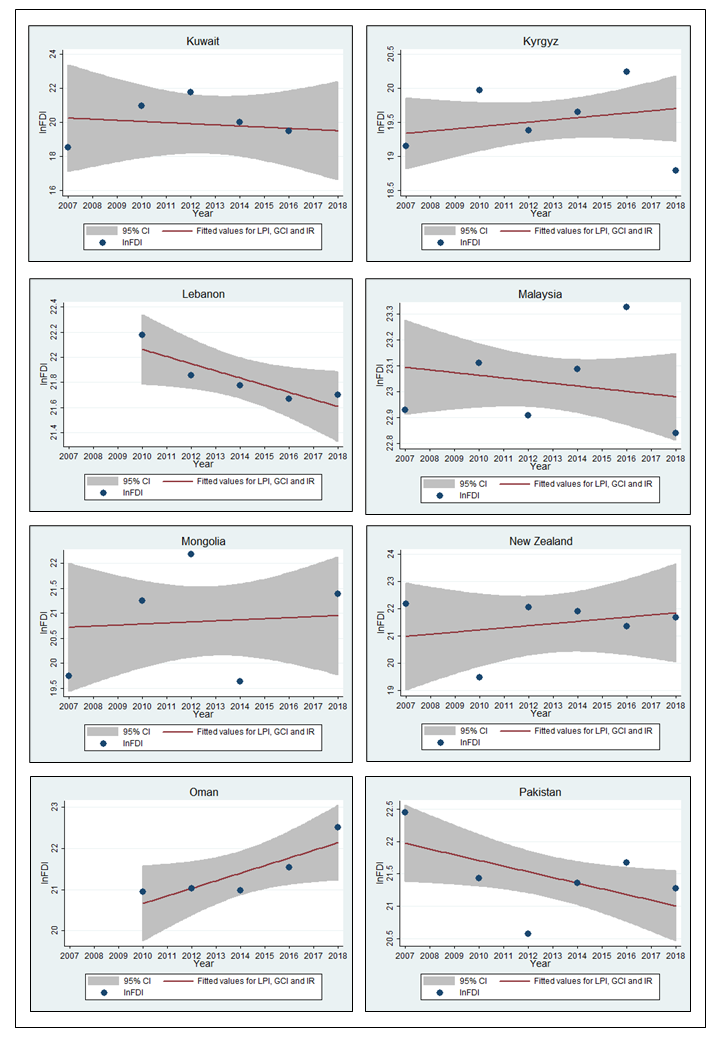


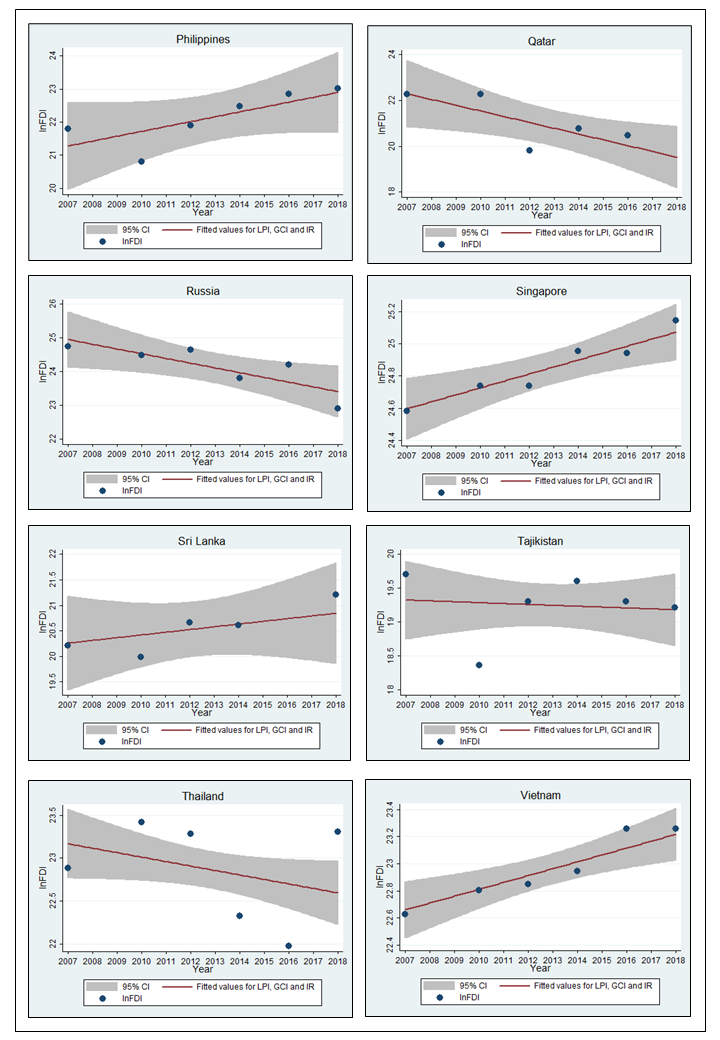


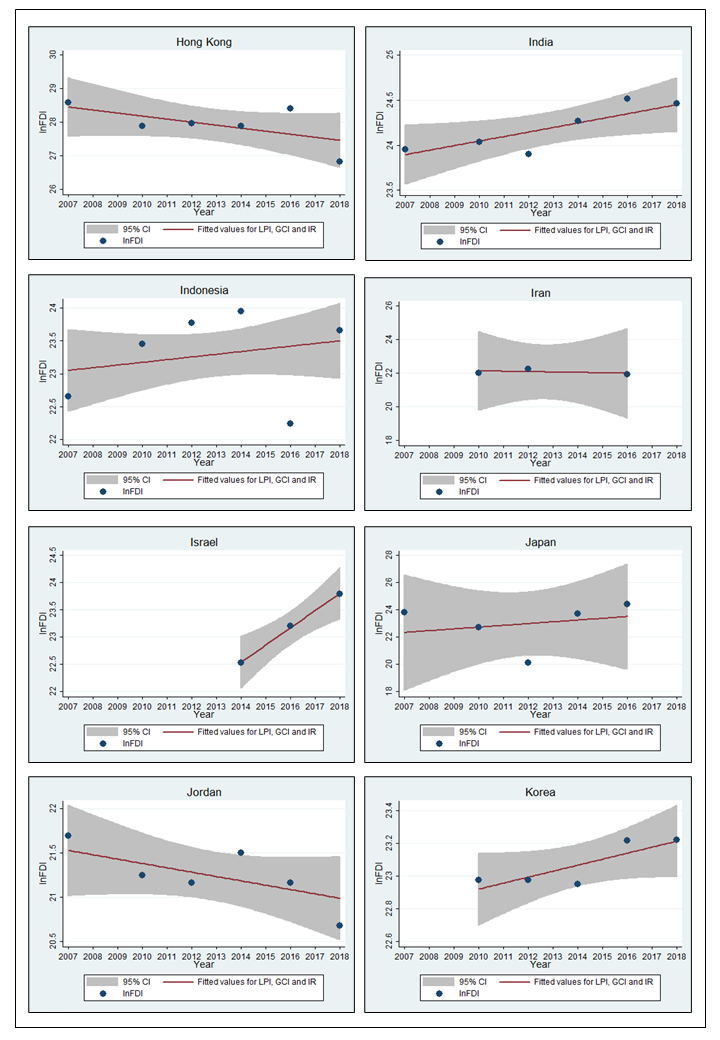


Source: Authors’ creation

Supplement: S4 Appendix — (DOCX) [file pone.0281246.s004.docx]
