# Supplementary material for: Determining the influence of LPI, GCI and IR on FDI: A study on the Asia and Pacific Region
Source: PLoS One. 2023 Feb 1;18(2):e0281246. doi: 10.1371/journal.pone.0281246 (PMC9891520; doi:10.1371/journal.pone.0281246)
Supplement: Attachment — Submitted filename: PLOS Review Comments.docx [file pone.0281246.s005.docx]

Dear Editor,

**Title:** **Determining the Influence of LPI, GCI and IR on FDI: A Study on Asia and Pacific Region**

**Originality:** The study lacks the originality to be justified. It lacks generalization aspects of the research study as a number of other variables might be added to enhance the quality of findings. Needs valid justification.

**Literature Review:** It lacks synthesis to build up the narration and relationship among the variables, under study. Provision of relevant and justified synthetic relationship of the variables in light of empirical literature along with theoretical background is most desirable.

**Methodology:** In methodology section and subsection of data is lacking proper justification of sub-variables explaining. The methodology is lacking valid literary reasoning. Justification for selection data set; why the six years data of 2007,2010,2012,2014, 2016 and 2018, missing the between years? Why some countries have been excluded within the sample size? Econometric techniques applied, needs further filtration and empirical justification in light of literature.

**Variables:** Why some key variables like Quality of Governance, Tax treaties and tax system, Balance of Payment, Exchange Rate etc. Provide valid justification in light of literature.

**References:** Do cite some good empirical papers. Provide valid empirical academic referencing.

**Grammar and Language:** It is advised to rephrase the text through the paper, improve the language and do a thorough proof read.

**Recommendation:**

The paper is only accepted if the following **major revisions** are made and addressed.

**Suggested Research Papers:**

Some of the relevant papers to be read and cited are as follows:

Shah, M. H. (2014). The significance of infrastructure for FDI inflow in developing countries. Journal of Life Economics, 1(2), 1-16.

Shah, M. H., & Khan, Y. (2016). Trade liberalization and FDI inflows in emerging economies. Shah, MH, & Khan, Y.(2016). Trade Liberalization and FDI Inflows in Emerging Economies. Business & Economic Review, 8(1), 35-52.

Shah, M. H., & Afridi, A. G. (2015). Significance of good governance for FDI inflows in SAARC countries. Shah, MH, & Afridi, AG (2015). Significance of Good Governance for FDI Inflows in SAARC Countries. Business & Economic Review, 7(2), 31-52.

Shah, M. H. (2017). The effect of macroeconomic stability on inward FDI in African developing countries. Shah, MH,(2016). The effect of macroeconomic stability on inward FDI in African developing countries. International Journal of Business Studies Review, 1(1), 1-11.

Ullah, Z., Shah, M. H., Khan, W., & Ali, A. (2021). Macroeconomic Factors As Drivers Of Foreign Portfolio Investment In Emerging Economy. Multicultural Education, 7(6).

Etc.

### Major Revisions

The research paper is only recommended with major revisions only if capitalized. These include:

1) Improve originality of the study by citing latest, most relevant and high impact factor research papers in the field.
2) The study has investigated low quality data as the author has mentioned himself; it creates the problem of originality. It is recommended to improve the data and data variables.
3) Literature review needs proper synthesis to explain the research gap, research question, hypothesis, variables selection and their relationship and the adoption of empirical techniques.
4) The research design do not seems appropriate to address the research question. Its suggested that either adopt a better research design or convey the research question more clearly.
5) Data variables need proper justification of selections and how they address the research problem?
6) The language needs a lot of improvement (grammatically and synthetically). A thorough proof read is recommended.
7) The recommendations given are too general but the study claims itself to be specific. It needs further elaboration. Supportive justification is need for the findings and conclusion in light of some good research studies.
8) Practical implications do not have something innovative and appealing. It might be improved with the increase of data sample, data quality and empirical tests.

In my opinion, if authors fulfill the above mentioned comments and suggestions, then the manuscript would be considered. After carefully review of manuscript, the studied paper fall in the category of major revision.

Good luck with your revisions.
